# Vibration-Based In-Situ Detection and Quantification of Delamination in Composite Plates

**DOI:** 10.3390/s19071734

**Published:** 2019-04-11

**Authors:** Hanfei Mei, Asaad Migot, Mohammad Faisal Haider, Roshan Joseph, Md Yeasin Bhuiyan, Victor Giurgiutiu

**Affiliations:** 1 Department of Mechanical Engineering, University of South Carolina, 300 Main Street, Columbia, SC 29208, USA; amigot@email.sc.edu (A.M.); haiderm@email.sc.edu (M.F.H.); rjoseph@email.sc.edu (R.J.); victorg@sc.edu (V.G.); 2 Department of Mechanical Engineering, College of Engineering, Thi-Qar University, Nasiriyah 64001, Iraq; 3 Collins Aerospace (A United Technologies Company), 100 Panton Rd, Vergennes, VT 05491, USA; mdyeasin.bhuiyan@utas.utc.com

**Keywords:** structural health monitoring, piezoelectric wafer active sensors, composites, delamination, local vibration, measured operational vibration shape

## Abstract

This paper presents a new methodology for detecting and quantifying delamination in composite plates based on the high-frequency local vibration under the excitation of piezoelectric wafer active sensors. Finite-element-method-based numerical simulations and experimental measurements were performed to quantify the size, shape, and depth of the delaminations. Two composite plates with purpose-built delaminations of different sizes and depths were analyzed. In the experiments, ultrasonic C-scan was applied to visualize the simulated delaminations. In this methodology, piezoelectric wafer active sensors were used for the high-frequency excitation with a linear sine wave chirp from 1 to 500 kHz and a scanning laser Doppler vibrometer was used to measure the local vibration response of the composite plates. The local defect resonance frequencies of delaminations were determined from scanning laser Doppler vibrometer measurements and the corresponding operational vibration shapes were measured and utilized to quantify the delaminations. Harmonic analysis of local finite element model at the local defect resonance frequencies demonstrated that the strong vibrations only occurred in the delamination region. It is shown that the effect of delamination depth on the detectability of the delamination was more significant than the size of the delamination. The experimental and finite element modeling results demonstrate a good capability for the assessment of delamination with different sizes and depths in composite structures.

## 1. Introduction

Composite materials have been extensively used in aerospace structures due to their high specific strength and stiffness, resistance to corrosion, light weight, and design flexibility [1]. However, damage of composite materials are more critical than those in metallic materials. Various damage modes exist (e.g., delamination, fiber fracture, and matrix cracking) and their detection and characterization are rather difficult. Delamination is the most common and dangerous failure mode for composites, because it takes place and grows in the absence of any visible surface damage, making it difficult to detect by visual inspection [2,3,4]. Due to the general anisotropic behavior [5] and complex damage scenarios, the successful implementation of delamination detection in aerospace composite structures is always challenging.

Various non-destructive testing (NDT) techniques have been developed for delamination detection in composite structures, including thermography, X-ray, and ultrasound methods [6,7]. However, these conventional methods are often costly, labor-intensive, and depend heavily on the skill and experience of the operator. Structural health monitoring (SHM) technologies offer a promising alternative and involve the continuous monitoring of a structure using integrated sensors [8,9,10,11,12]. In recent decades, many SHM techniques have been developed for delamination detection, such as electromechanical impedance (EMI) [13,14], acoustic emission (AE) [15,16], and guided-wave-based methods [17,18,19,20,21,22,23,24,25,26,27,28].

Although numerous research work have been dedicated to the above-mentioned SHM methods, the focus of this paper is on a vibration-based method for delamination detection. The idea behind vibration-based damage detection is that damage causes a change in the local stiffness, mass, and structural damping, which results in recognizable changes in vibration characteristics such as natural frequencies, mode shapes, and modal damping. A comprehensive literature review on vibration-based damage detection methods was implemented in reference [29]. Various vibration-based delamination detection approaches have been developed in the literature by many authors [30,31,32,33,34,35]. Shang et al. [30] proposed a model-based method in the delamination detection of composite plates using modal testing and a subset selection technique. Klepka et al. [31] used the nonlinear vibro-acoustic responses of a composite plate for impact-induced delamination detection through a scanning laser Doppler vibrometer (SLDV) measurement. Ooijevaar et al. [32] used the modal curvature for delamination detection in composite skin-stiffener structures using SLDV measurement. Garcia et al. [33] investigated a delamination detection method based on a multivariate statistical procedure and the time domain structural vibration response. Delamination detection based on the wavelet packet transform of vibration responses using sparse sensing piezoelectric transducers was experimentally investigated by Gaudenzi et al. [34]. Recently, Yang and Oyadiji [35] numerically investigated the delamination detection in composite laminates using a modal frequency surface by attaching a point mass at different locations.

Vibration-based methods using mode shapes to detect damage require the accurate measurement of displacement responses. The scanning laser Doppler vibrometer has been widely used for vibration response measurements [30,31,32]. For the actuators, various methods of exciting the monitored structures can be found in the literature, such as shakers [36,37,38], hammers [39], and speakers [40]. Moreover, several researchers have explored the capability of piezoelectric wafer active sensors (PWAS) transducers for vibration-based delamination detection [30,41,42,43]. The main advantage of PWAS transducers over conventional ultrasonic probes is their low cost and light weight [17]. They can be permanently bonded on the host structures in large quantities and achieve the real-time monitoring of the structural health status. However, only low-frequency (modal) excitation using PWAS transducers has been conducted in the above-mentioned literature. To overcome this issue, the concept of local defect resonance, as first described by Solodov in [44], has recently gained considerable attention for the detection of delamination in composites [45,46,47,48,49,50]. High-frequency excitations were used to obtain a localized resonant activation of the defected zones [45]. The local defect resonance is the interaction of ultrasonic waves with the damaged region at a frequency that matches the local defect resonance, which results in a significant increase of the vibration amplitude only in the localized damaged region [46]. These studies facilitate the understanding of local vibration-based SHM applications.

**Figure 1:**
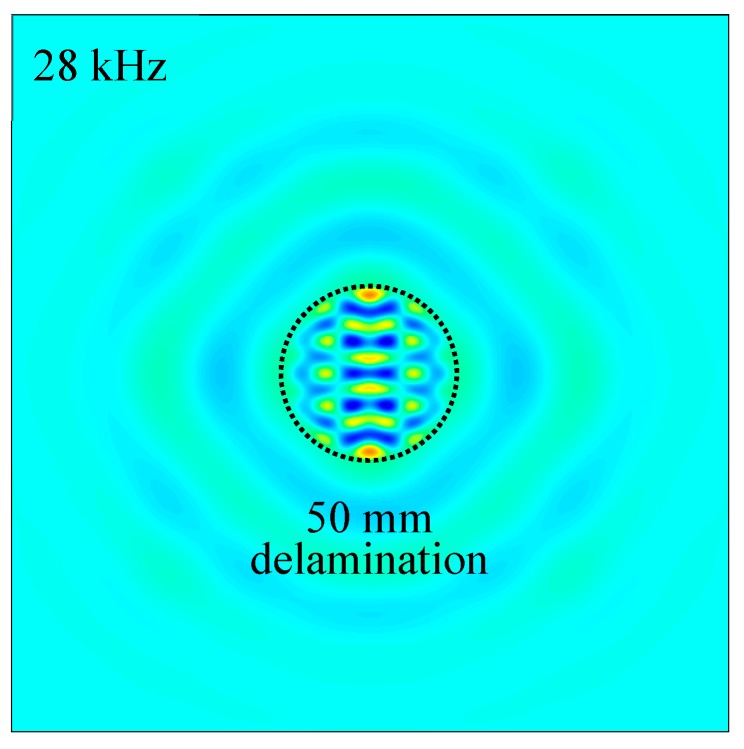
Example of using high-frequency local vibration to detect internal delamination in a laminated composite: 50 mm delamination excited at 28 kHz displays a clear local vibration mode that indicates the delamination size.

In this paper, a new methodology is presented to detect and quantify the size, shape, and depth of delamination in composite structures based on the high-frequency local vibration under PWAS transducer excitation, as exemplified in Figure 1. In this method, a PWAS transducer is used for the high-frequency excitation through a linear sine wave chirp. Measured operational vibration shapes of the composite plate at the resonance frequencies of delaminations are extracted and utilized to detect and quantify the delamination. The effects of size and depth of delamination on the measured operational vibration shapes are investigated. First, ultrasonic C-scan is used to detect and visualize the purpose-built delaminations in two composite plates. Then, the local defect resonance frequency corresponding to the resonance frequency of delamination is determined using the SLDV measurements on a cross-ply composite plate with delaminations of various sizes and a unidirectional composite plate with delaminations of various depths. Next, multi-physics three-dimensional (3D) finite element (FE) modeling is used to find the operational vibration shapes at resonance frequencies. Finally, the measured operational vibration shapes extracted from the SLDV measurements are used to detect and quantify the delaminations in composite plates.

## 2. Composite Specimens

We manufactured two in-house composite plates with different delaminations and stacking sequences. In the present study, six cases of delaminations of different sizes and depths were investigated. 

### 2.1. Cross-Ply Composite Plate

**Figure 2:**
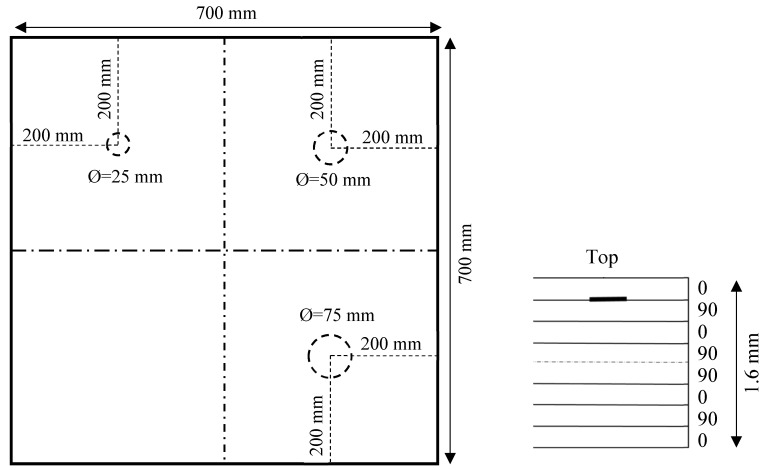
Schematic of a 1.6 mm cross-ply [0/90]_2s_ carbon fiber-reinforced polymer (CFRP) composite plate with three simulated delaminations of various sizes at the same depth.

The first specimen was a 1.6 mm thick in-house cross-ply carbon fiber-reinforced polymer (CFRP) composite plate with a stacking sequence of [0/90]_2s_. Three simulated delaminations were generated by inserting Teflon films of different sizes between the first ply and second ply before curing in the autoclave. The sizes of simulated circular delaminations were 25, 50, and 75 in diameter, respectively. Figure 2 shows a schematic of the composite plate with simulated delaminations. The dimensions of the specimen were 700 mm × 700 mm × 1.6 mm. The material properties were measured experimentally using the ultrasonic immersion technique as described in [5], given in Table 1.

### 2.2. Unidirectional Composite Plate

**Figure 3:**
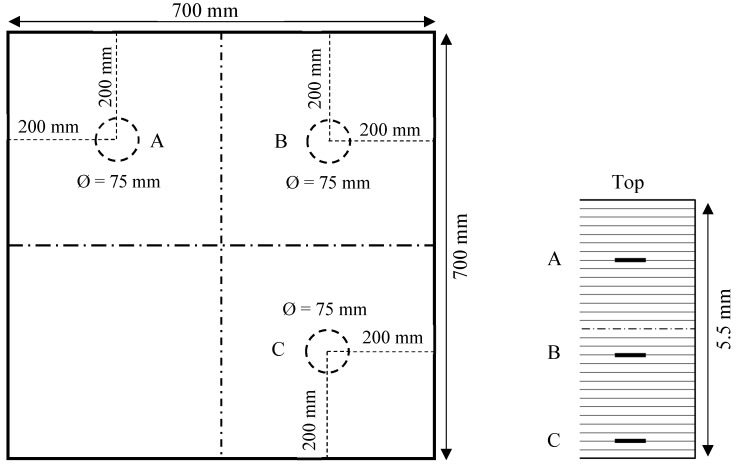
Schematic of the 5.5 mm unidirectional [0]_30_ CFRP composite plate with three equally sized delaminations at various depths.

The second specimen was a 5.5-mm-thick in-house unidirectional CFRP composite plate with a stacking sequence of [0]_30_. The configuration of the unidirectional composite plate is given in Figure 3. The dimensions of the specimen were 700 mm × 700 mm × 5.5 mm. The unidirectional prepreg was the same as the first specimen. This specimen had three simulated delaminations at different depths across the plate thickness, which were created by inserting three circular Teflon films of the same size (75 mm in diameter). Delamination A (near the top surface) was created between plies 7 and 8. Similarly, delamination B (near the mid-plane) was generated between plies 18 and 19, and delamination C (near the bottom surface) was made between plies 28 and 29, as shown in Figure 3.

## 3. Ultrasonic Non-Destructive Testing (NDT) of the Composite Specimens

**Figure 4:**
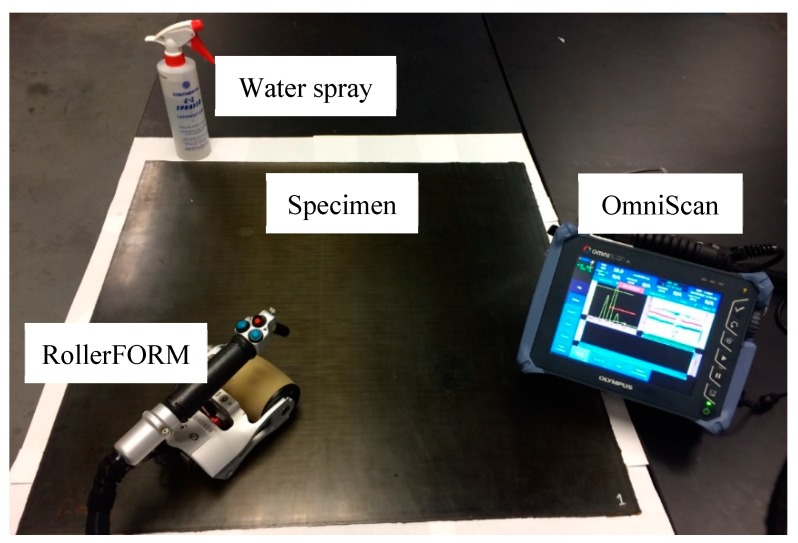
Experimental setup of the ultrasonic detection of the composite specimens.

In this section, ultrasonic non-destructive testing (NDT) was performed on the cross-ply and unidirectional composite plates. The purpose of the NDT was to detect and verify the simulated delaminations. The Olympus RollerFORM [51], a new phased array wheel probe, was used to inspect the specimen. In the experiment, a 3.5 MHz phased array probe was used to detect the delaminations. The coverage width of the probe was approximately 49 mm. Therefore, a large inspection area could be divided into several area scans. The width of each area scan was 49 mm. The experimental setup of the NDT detection on the composite plates is shown in the Figure 4. The water spray was the couplant facilitating the transmission of ultrasonic energy from the RollerFORM into the test specimen. The OmniScan collected and processed the received signals from the RollerFORM. It could create A-scan plot, B-scan, and C-scan images of the inspected area.

### 3.1. NDT Detection on the Cross-Ply Composite Plate

**Figure 5:**
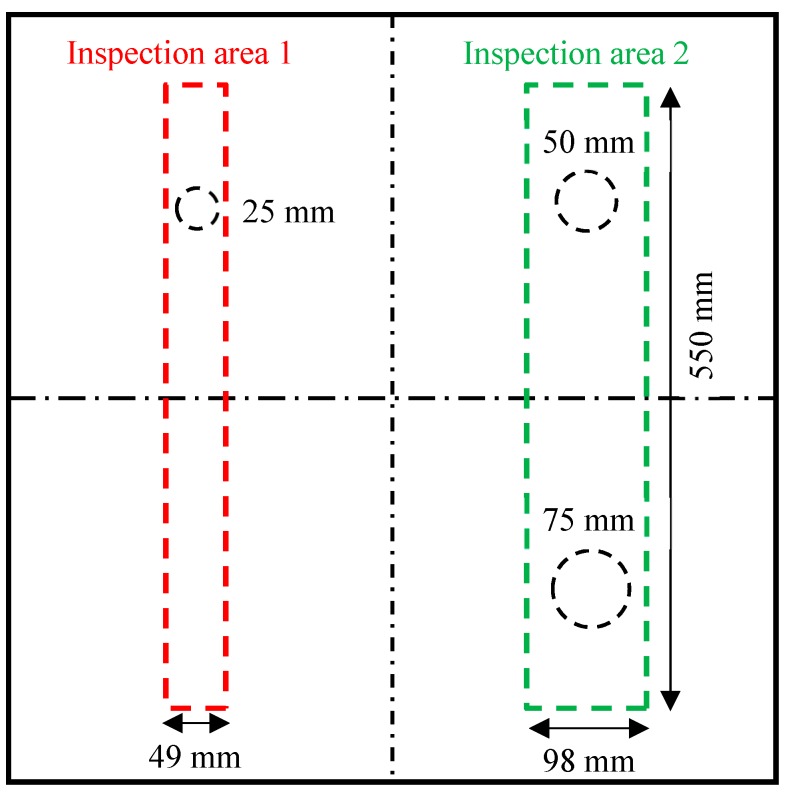
Schematic of non-destructive testing (NDT) inspection areas on the cross-ply [0/90]_2s_ CFRP composite plate.

Two inspection areas were conducted on the cross-ply CFRP composite plate to cover all the three delaminations, as shown in Figure 5. The first inspection area (550 mm × 49 mm) had the pristine area and a 25 mm delamination area. It only required one area scan because its width was equal to the width of the phase array beam (49 mm). The second inspection area was 550 mm × 98 mm, covering the 50 mm delamination and the 75 mm delamination. This area was divided into two area scans. 

**Figure 6:**
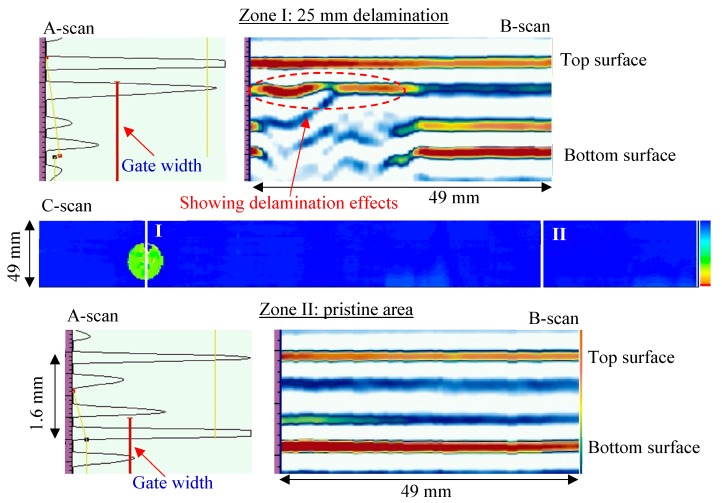
NDT results of the first inspection area on the cross-ply [0/90]_2s_ CFRP composite plate: pristine area and 25 mm delamination.

Figure 6 shows the NDT results of the first inspection area, including the A-scan plot, B-scan image, and C-scan image. The C-scan image shows the presence of small circular delamination (25 mm in diameter). The location of the delamination across the thickness could be determined based on the color map. The section view of Zone I (25 mm delamination) shows that the delamination was close to the top surface. The vertical red lines in the A-scan plots represent the gate width that must cover the back-wall signals. The A-scan plot shows that the reflected signal of delamination had a strong amplitude, whereas the back-wall signal was very weak because the delamination blocked the phase array beam to hit the bottom surface of the plate. However, no strong reflected signal between the top and bottom surfaces could be observed from the section view of the pristine area (Zone II).

**Figure 7:**
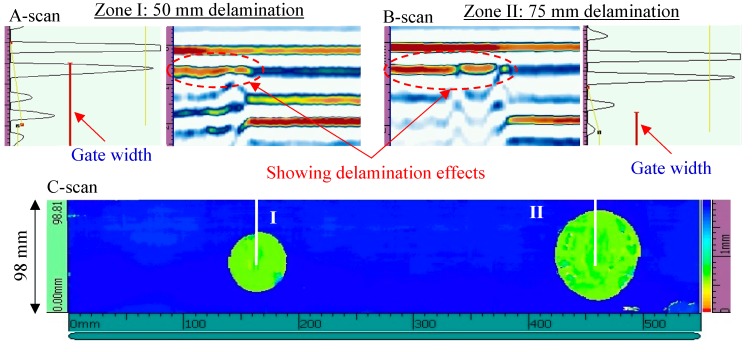
NDT results of the second inspection area on the cross-ply [0/90]_2s_ CFRP composite plate: 50 mm delamination and 75 mm delamination.

The NDT results of the second inspection area are shown in Figure 7. From the C-scan result, two delaminations of different sizes (50 mm and 75 mm in diameter) could be clearly observed. These delaminations had the same depths across the thickness because they had the same color map. The section views of Zone I and Zone II show the strong delamination effects, where the weak back-wall signals could be also observed.

### 3.2. NDT Detection on the Unidirectional Composite Plate

**Figure 8:**
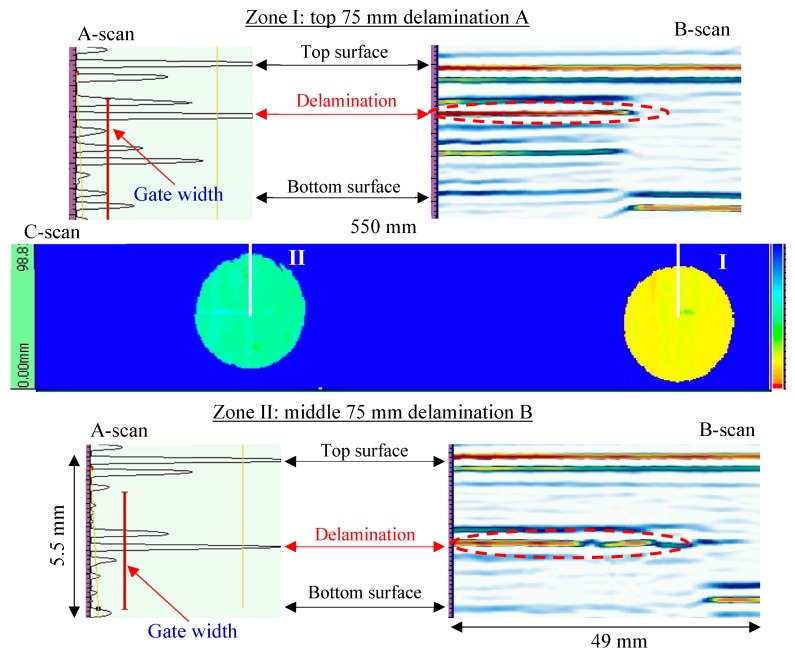
NDT results of the first inspection area on the unidirectional [0]_30_ CFRP composite plate: top and middle 75 mm delaminations.

For the 5.5-mm-thick unidirectional [0]_30_ CFRP composite plate, two inspection areas of the same size (550 mm × 98 mm) were conducted. The first inspection area had delamination A (near the top surface) and delamination B (near the mid-plane), while the second inspection area only included the delamination C (near the bottom surface). The NDT results of the first inspection area are shown in Figure 8. From the C-scan image, two circular delaminations of the same size with different color maps can be observed, which means that they had different depths. The B-scan image of the top and middle delaminations indicates the depths across the plate thickness. The A-scan plot shows that the reflected signals of the delaminations had a strong amplitude while the back-wall signal was weak because it was under the delamination. 

**Figure 9:**
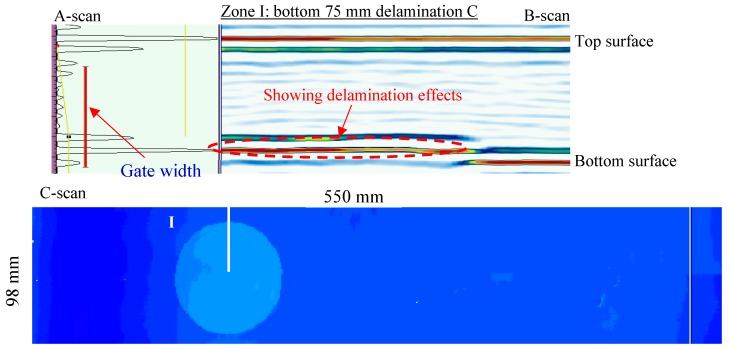
NDT results of the second inspection area on the unidirectional [0]_30_ CFRP composite plate: bottom 75 mm delamination.

The NDT results of the second inspection area on the unidirectional [0]_30_ CFRP composite plate are shown in Figure 9. The C-scan image shows that the circular delamination C had a light blue color map, which means that the delamination was close to the bottom surface. The section view of Zone I demonstrates that the delamination C was close to the bottom surface. Therefore, the sizes and depths of these delaminations could be quantified from the NDT detection, which is consistent with the design.

## 4. Scanning Laser Doppler Vibrometer (SLDV) Measurements on the Composite Specimens

### 4.1. SLDV Measurement on the Cross-Ply Composite Plate

**Figure 10:**
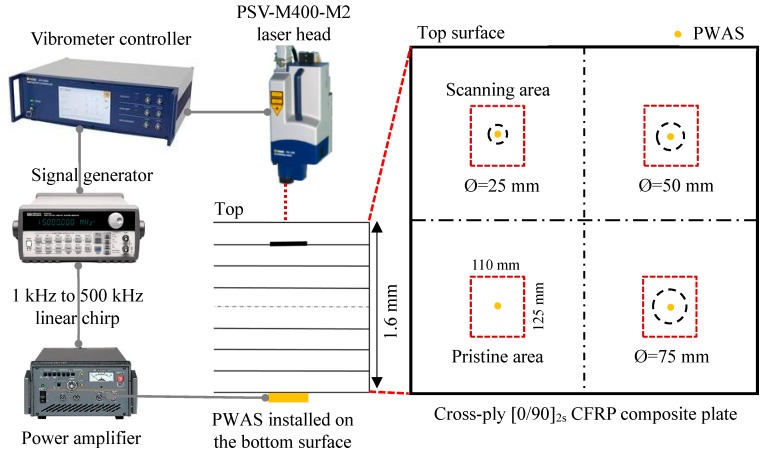
Experimental setup on the cross-ply [0/90]_2s_ CFRP composite plate using the scanning laser Doppler vibrometer (SLDV) measurement. PWAS: piezoelectric wafer active sensors.

In this experiment, scanning laser Doppler vibrometer measurement was implemented to study the local vibration of four areas (one pristine and three damaged areas), as shown in Figure 10. The size of each scanning area was 110 mm × 125 mm. At the center of each inspection area, a circular PWAS transducer (APC 850, 7 mm diameter and 0.2 mm thick) was bonded on the bottom surface as the excitation source. 

A function generator was used to generate a linear sine wave chirp from 1 to 500 kHz, with a duration of 1 ms. This chirp excitation signal was amplified to 140 Vpp by the power amplifier and applied to the PWAS transducers for the high-frequency excitation. The wavefield data were recorded by a Polytec PSV-400-M2 scanning laser Doppler vibrometer (SLDV) on the top surface of the plate. To avoid aliasing, a high sampling rate of 50 MHz was used to collect the response signals, which is 100 times the highest frequency (500 kHz) in the response signal. The out-of-plane velocity and the local vibration of the specimen were measured and visualized by the SLDV. Reflective tapes were used to improve the signal quality on the scanning area. To ensure the accuracy of the experimental results, multiple trials were conducted before recording the final data. The response signals were insensitive to experiments performed on different days and good repeatability of experimental data was achieved by comparing the response signals of different days at the same measurement point.

**Figure 11:**
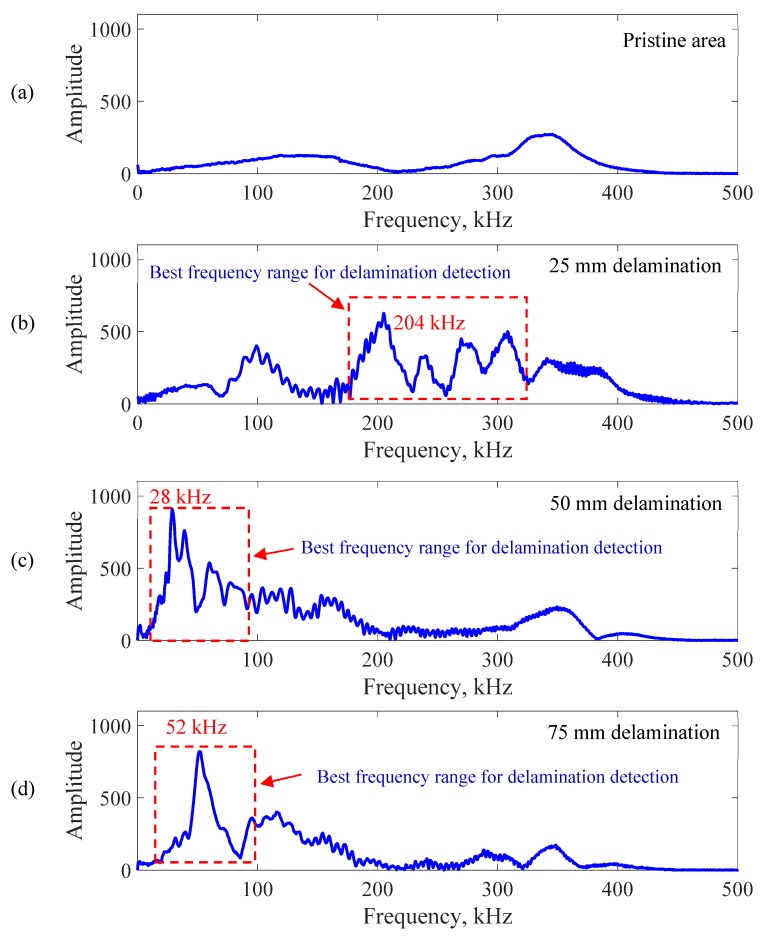
Frequency spectrum of the response signals at the center point of different scanning areas: (**a**) pristine area; (**b**) 25 mm delamination; (**c**) 50 mm delamination; (**d**) 75 mm delamination.

A fast Fourier transform (FFT) of the response signals was performed to determine the local defect resonance frequencies. The zero-padding response signal in the FFT analysis was used to reduce the leakage phenomenon. Figure 11 shows the frequency spectrum of measured response signals at the center point of the four scanning areas. Figure 11a shows the FFT result of the pristine area. Figure 11b–d show the FFT results of the response signals at the center point of the 25, 50, and 75 mm delaminations, respectively. The delamination significantly modified the frequency response compared with the results of the pristine area. 

The local defect resonance frequencies could be obtained by peak picking from the delaminations’ FFT results. More specifically, some resonance peaks in the frequency range from 80 to 350 kHz were found for the 25 mm delamination. However, the resonance peaks of the 50 mm and 75 mm delaminations were below 100 kHz. The delamination size had a significant effect on the local defect resonance frequency. These resonance frequencies of the different delamination sizes are summarized in Table 2. The measured operational vibration shapes at these local defect resonance frequencies were extracted from the SLDV area measurement, which is used to quantify the delaminations of different sizes in Section 6.

### 4.2. SLDV Measurement on the Unidirectional Composite Plate

**Figure 12:**
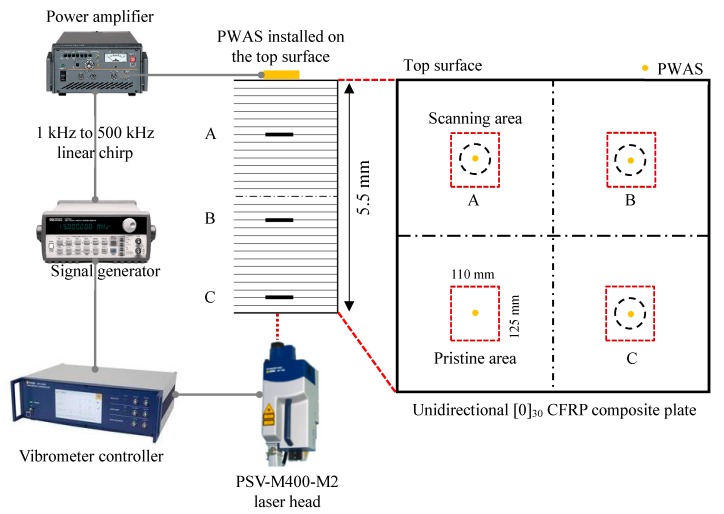
Experimental setup on the unidirectional [0]_30_ CFRP composite plate using the scanning laser Doppler vibrometer (SLDV) measurement.

The experimental setup of the SLDV measurements on the unidirectional [0]_30_ CFRP composite plate was similar to the first specimen in Section 4.1. Four areas (one pristine and three damaged areas) were inspected to investigate the local vibration, as shown in Figure 12. The size of each scanning area was 110 mm × 125 mm. In this experiment, at the center of each inspection area, a circular PWAS transducer (APC 850, 7 mm diameter and 0.2 mm thick) was bonded on the top surface as the excitation source. The same linear sine wave chirp from 1 to 500 kHz was applied to the PWAS transducers for the high-frequency excitation. The wavefield data were recorded by the SLDV on the bottom surface of the plate.

**Figure 13:**
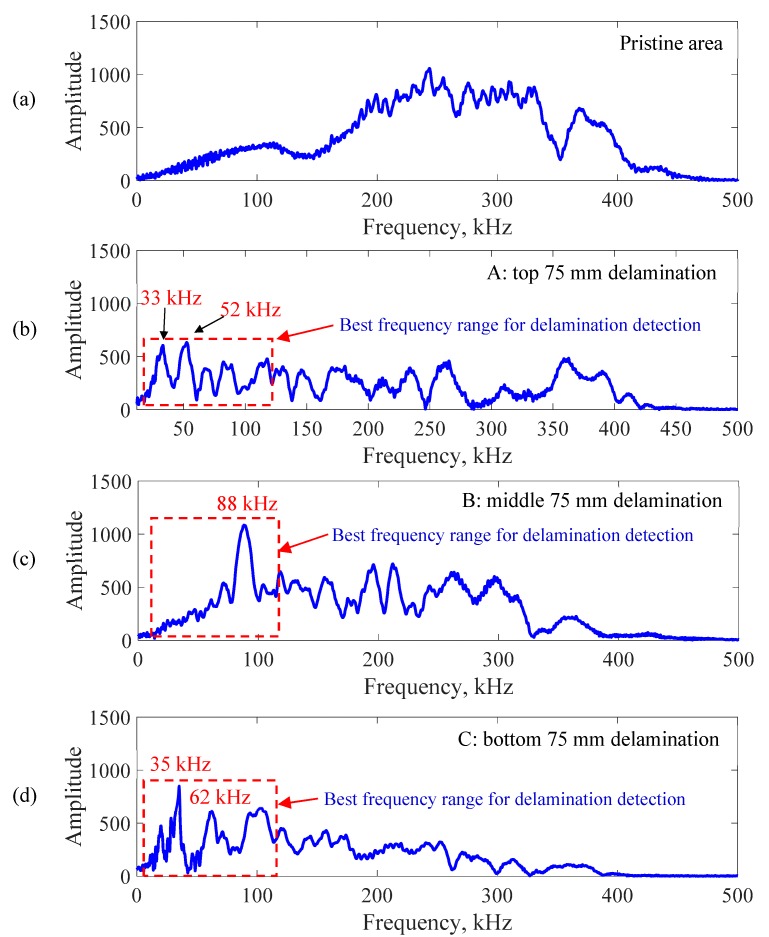
Frequency spectrum of the response signals at the center point of different scanning areas: (**a**) pristine area; (**b**) top 75 mm delamination A; (**c**) middle 75 mm delamination B; (**d**) bottom 75 mm delamination C.

A fast Fourier transform (FFT) of the response signals was performed to find the local defect resonance frequencies of the delaminations at different depths. Figure 13 shows the frequency spectrum of measured response signals at the center point of the four scanning areas. Figure 13a shows the FFT of the response signal for the pristine area. Figure 13b–d show the FFT results of the response signals at the center point of the top, middle, and bottom 75 mm delaminations, respectively. The delaminations at various depths strongly changed the frequency response compared with the result of the pristine area. The local defect resonance frequencies could be obtained by peak picking from the FFT results. The resonance frequencies of the delaminations at different depths are given in Table 3. The corresponding measured operational vibration shapes at these local defect resonance frequencies are extracted from the SLDV area measurement for the quantification of the delaminations at different depths in Section 6.

## 5. Multi-Physics Finite Element Modeling

In this section, the multi-physics finite element (FE) modeling of the two composite plates was conducted to extract the measured operational vibration shapes of the local vibration.

### 5.1. Finite Element Model for the Cross-Ply Composite Plate

Three-dimensional (3D) finite element models were utilized to simulate the 1.6-mm-thick eight-ply [0/90]_2s_ CFRP composite plate with delaminations of different sizes. Four different local FE models were implemented to simulate the local vibrations of the pristine area: 25 mm, 50 mm, and 75 mm delaminations, respectively. The dimensions of each FE model were 200 mm × 200 mm × 1.6 mm. Non-reflective boundary (NRB) developed by Shen and Giurgiutiu [52] can eliminate boundary reflections, and thus allow for the simulation in an infinite medium with small-size models. This NRB was implemented around the FE models to calculate the steady-state response under PWAS transducer excitation with sinusoidal waves. This analysis can be performed for any specific frequency of interest. 

**Figure 14:**
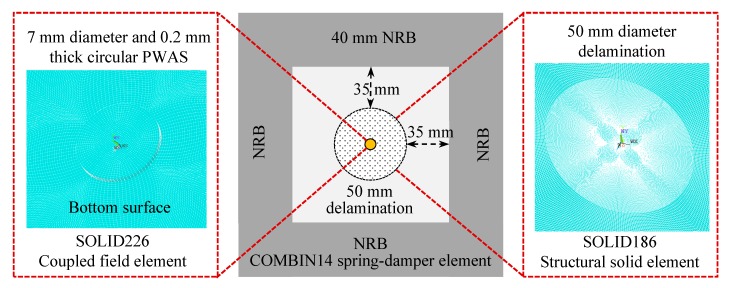
Multi-physics local finite element model of the cross-ply [0/90]_2s_ CFRP composite plate with 50 mm delamination. NRB: non-reflective boundary.

Figure 14 shows the FE model of the cross-ply composite plate with 50 mm delamination as a representative. The delamination was created between plies 1 and 2 by specifying the delamination as two planes, defined by the same coordinates but not tied together. The PWAS transducer was modeled with coupled field hexahedral elements (SOLID226) in the commercial finite element package ANSYS 17.0, which couple the electrical and mechanical variables. 3D structural solid hexahedral elements (SOLID186) with quadratic shape function interpolation were used to mesh the composite plate. COMBIN14 spring-damper elements were used to construct the 40-mm NRB around the model. A Rayleigh damping (*β* = 3 × 10^−8^) was considered to simulate the damping effect of the composite material. The damping value was determined by iterative calibration to match the experimentally measured attenuation coefficient. A comparable value was used in References [53,54]. The stiffness proportional damping coefficient *β* introduces damping proportional to the strain rate, which can be thought of as damping associated with the material itself. The material properties of the composite plate are given in Table 1. The APC-850 material properties were assigned to the PWAS transducer as described in Gresil and Giurgiutiu [53].

(1) $$\left[C_{p}\right]=\left[\begin{array}{cccccc} 97 & 49 & 44 & 0 & 0 & 0\\ 49 & 97 & 44 & 0 & 0 & 0\\ 44 & 44 & 84 & 0 & 0 & 0\\ 0 & 0 & 0 & 22 & 0 & 0\\ 0 & 0 & 0 & 0 & 22 & 0\\ 0 & 0 & 0 & 0 & 0 & 24 \end{array}\right]GPa,$$

(2) $$\left[\varepsilon_{p}\right]=\left[\begin{array}{ccc} 947 & 0 & 0\\ 0 & 947 & 0\\ 0 & 0 & 605 \end{array}\right]\times 10^{-8}F/m,$$

(3) $$\left[e_{p}\right]=\left[\begin{array}{cccccc} 0 & 0 & 0 & 0 & 12.84 & 0\\ 0 & 0 & 0 & 12.84 & 0 & 0\\ -8.02 & -8.02 & 18.31 & 0 & 0 & 0 \end{array}\right]C/m^{2},$$

where $\left[C_{p}\right]$ is the stiffness matrix, $\left[\varepsilon_{p}\right]$ is the dielectric matrix, and [*e_p_*] is the piezoelectric matrix. The density of the PWAS material was 7600 kg/m^3^.

The maximum acceptable element size to ensure convergence is *λ*_min_/*l_e_* ≥ 20 [55], where *λ*_min_ is the minimum wavelength and *l_e_* is the element size. In this study, the A0 mode had the minimum wavelength, which was around 10 mm. Therefore, the mesh size adopted was 0.5 mm for the in-plane direction and 0.2 mm for the thickness direction. The PWAS transducer and delamination regions were meshed with even smaller elements to guarantee that more than 30 elements existed per wavelength, which is substantially more than the minimum requirement of 20 elements. A total of 582,640 elements were used in this local FE model. 

**Figure 15:**
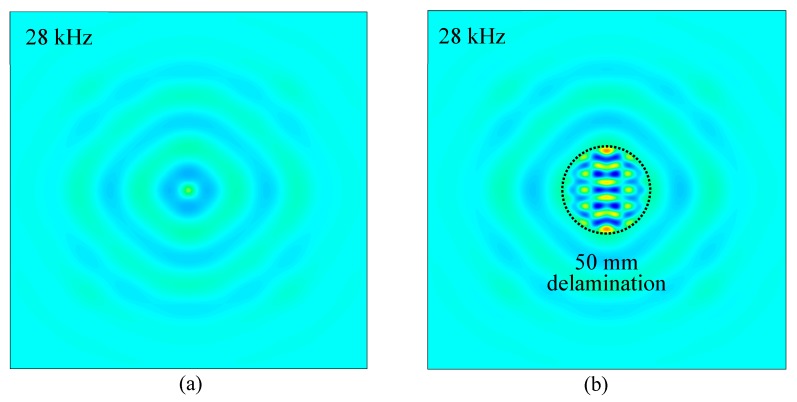
FEM-measured operational vibration shape comparison of the cross-ply composite plate between pristine area and 50 mm delamination at 28 kHz: (**a**) pristine area; (**b**) 50 mm delamination.

**Figure 16:**
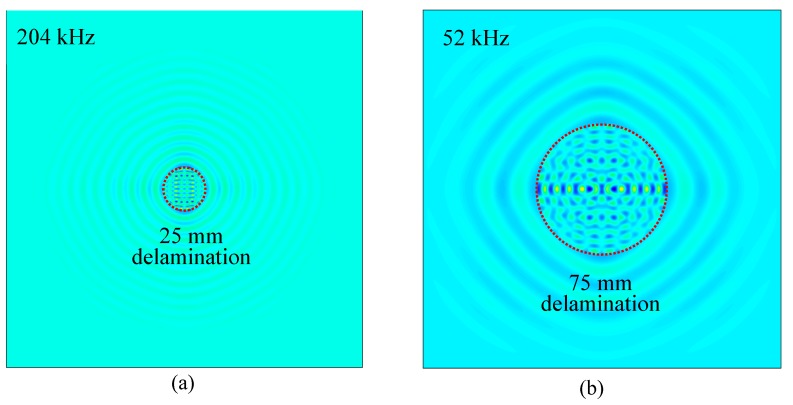
FEM-measured operational vibration shapes of the cross-ply composite plate at the resonance frequencies: (**a**) 25 mm delamination at 204 kHz; (**b**) 75 mm delamination at 52 kHz.

The measured operational vibration shapes at the local defect resonance frequencies obtained in Section 4.1 were extracted from the FEM harmonic analysis. Figure 15 shows the comparison of out-of-plane measured operational vibration shape between the pristine area and 50 mm delamination at the resonance frequency of 28 kHz. A strong local vibration only in the delamination region under the PWAS transducer excitation could be clearly observed. The shape and size of the delamination could be determined from the measured operational vibration shape. Similarly, the FEM-measured operational vibration shapes of 25 mm and 75 mm delaminations at the corresponding resonance frequencies are given in Figure 16. Clear local vibration shapes indicating the shape and size of the delamination were noted. Therefore, the measured operational vibration shapes could be used to quantify the shape and size of the delaminations.

### 5.2. Finite Element Model for the Unidirectional Composite Plate

In this section, 3D finite element models were utilized to simulate the 5.5-mm-thick thirty-ply [0]_30_ CFRP composite plate with delaminations at various depths. Four different local FE models were conducted to simulate the local vibrations of the pristine area and top, middle, and bottom delaminations, respectively. The dimensions of the FE models were 200 mm × 200 mm × 5.5 mm. A NRB was implemented around the FE models to analyze harmonic responses under PWAS transducer excitation with sinusoidal waves. The material properties of the PWAS transducer were the same as in Section 5.1.

**Figure 17:**
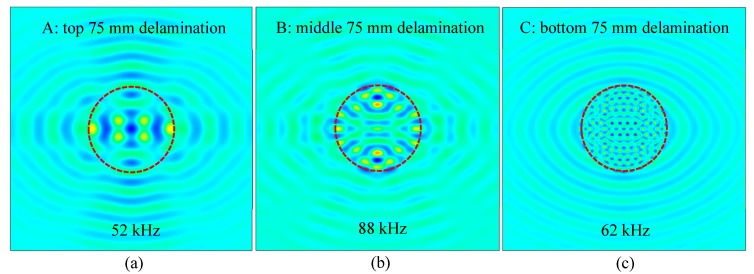
FEM-measured operational vibration shapes of delaminations at different depths in the unidirectional CFRP composite plate: (**a**) top 75 mm delamination at 52 kHz; (**b**) middle 75 mm delamination at 88 kHz; (**c**) bottom 75 mm delamination at 62 kHz.

Figure 17 shows the measured operational vibration shapes of delaminations at different depths in the unidirectional CFRP composite plate. In this FEM simulation the PWAS transducer was installed on the top surface to be consistent with the experimental setup in Section 4.2. The measured operational vibration shapes at different resonance frequencies were extracted at the bottom surface. A denser vibration pattern was observed when the delamination was close to the bottom surface, as shown in Figure 17. The depth of the delamination had a significant effect on pattern of the measured operational vibration shapes.

## 6. Vibration-Based Delamination Detection of Composite Plates

Previous studies have confirmed the multiple reflections within the delamination area under the PWAS transducer excitation and that a considerable amount of ultrasonic energy is trapped in the delaminated region to generate a strong local vibration [46]. Therefore, the delamination can be quantified by analyzing the measured operational vibration shape of the local vibration. The measured operational vibration shapes could be visualized at the resonance frequencies from the SLDV area scanning data in Section 4. First, fast Fourier transform (FFT) of all the response signals obtained in the SLDV measurements was implemented. Then, the measured operational vibration shape was generated using the FFT amplitudes at a certain resonance frequency of all the measurement points. 

### 6.1. Delamination Detection on the Cross-Ply Composite Plate

**Figure 18:**
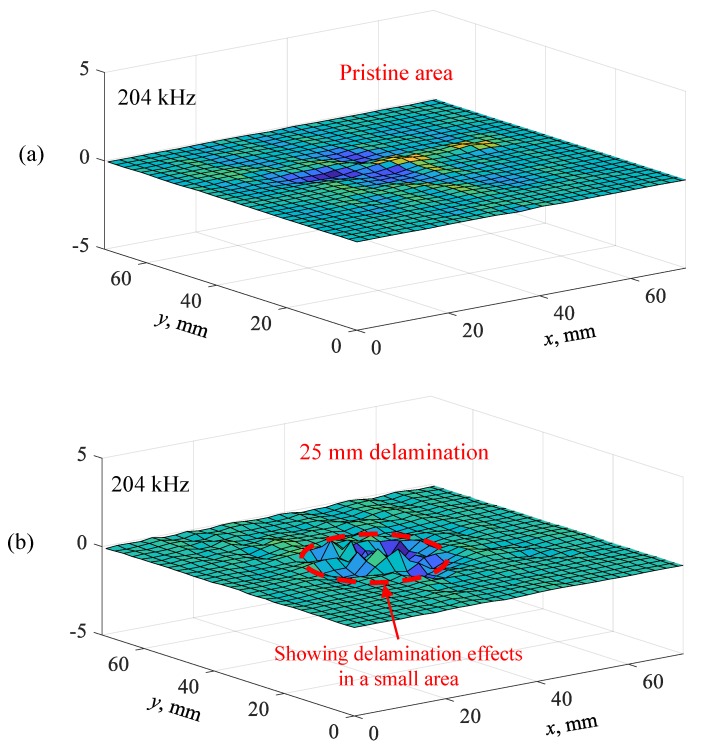
Comparison of measured operational vibration shapes on the cross-ply CFRP composite plate: (**a**) pristine area at 204 kHz; (**b**) 25 mm delamination at resonance frequency of 204 kHz.

In this section, the effect of delamination size on the measured operational vibration shape of the local vibration was investigated. The Fourier transform of all the response signals was implemented and the measured operational vibration shape was visualized using the FFT amplitudes of a certain frequency at all the measurement points. Figure 18 shows the comparison of measured operational vibration shapes between the pristine area and 25 mm delamination at the resonance frequency of 204 kHz. There was no clear local vibration on the pristine area, whereas a strong local vibration in a small area was observed for the 25 mm delamination. This is because a considerable amount of ultrasonic energy was trapped in the delaminated area. In addition, the measured operational vibration shape can indicate the size and shape of the delamination. Therefore, the shape and size of the delamination could be easily determined. 

**Figure 19:**
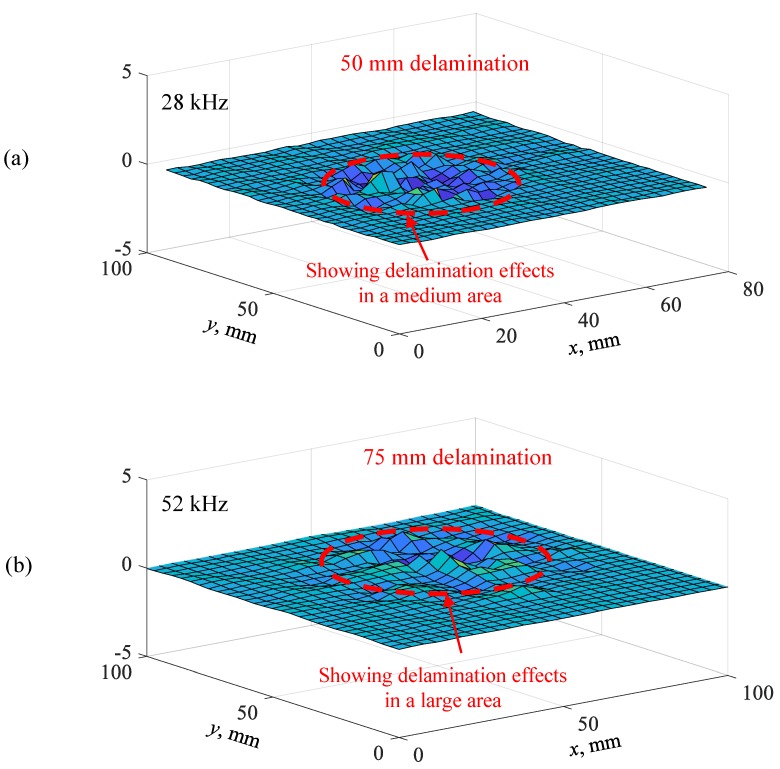
Measured operational vibration shapes on the cross-ply CFRP composite plate: (**a**) 50 mm delamination at 28 kHz; (**b**) 75 mm delamination at 52 kHz.

Figure 19a,b show the measured operational vibration shapes of the 50 mm delamination at 28 kHz resonance frequency and the 75 mm delamination at 52 kHz resonance frequency, respectively. Clear local vibrations with high amplitudes in the delamination areas were observed. For the 50 mm delamination, local vibrations in a medium area were observed, whereas large-area local vibrations were noted when the delamination size was 75 mm. These measured operational vibration shapes at resonance frequencies could be used directly to quantify the shape and size of the 50 mm and 75 mm delaminations. Therefore, the local vibration technique was successfully utilized to detect and quantify the delaminations of different sizes in the cross-ply CFRP composite plate.

### 6.2. Delamination Detection on the Unidirectional Composite Plate

**Figure 20:**
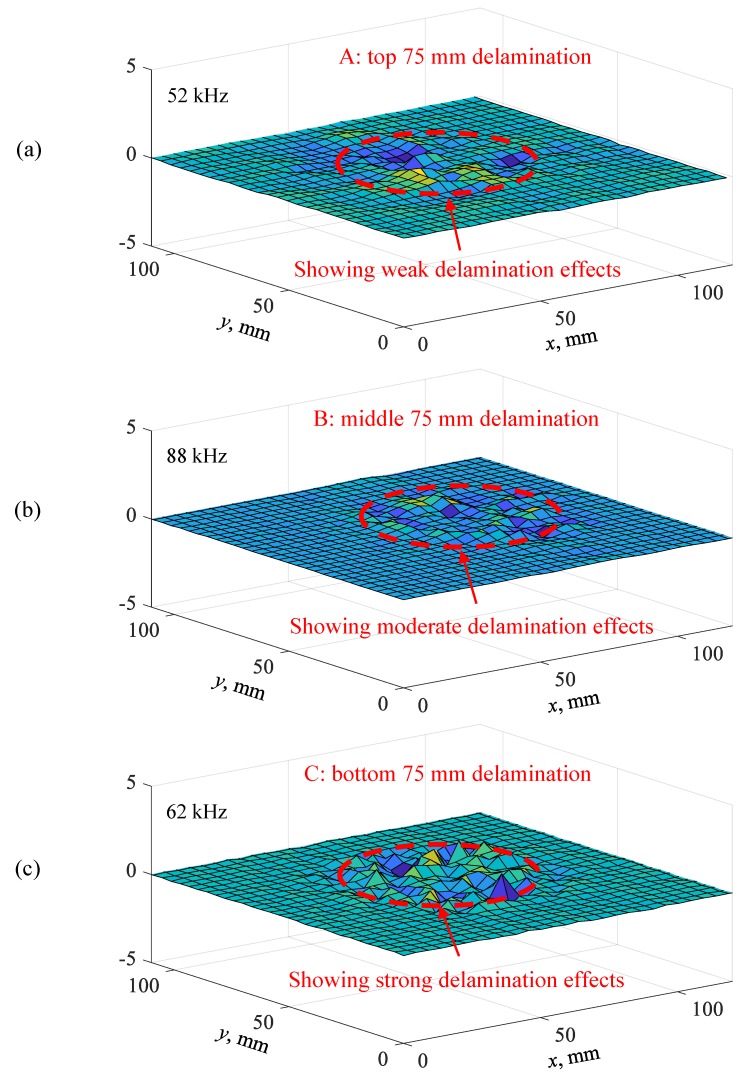
Measured operational vibration shapes of delaminations at different depths in the unidirectional CFRP composite plate: (**a**) top 75 mm delamination at 52 kHz; (**b**) middle 75 mm delamination at 88 kHz; (**c**) bottom 75 mm delamination at 62 kHz.

In this section, the effect of delamination depth on the measured operational vibration shape of local vibrations was studied. Figure 20 shows the measured operational vibration shape of delaminations at three different depths (top, middle, and bottom) in the unidirectional composite plate. For the top 75 mm delamination shown in Figure 20a, a weak delamination effect was observed when the delamination was away from the measuring surface. As shown in Figure 20b, a moderate delamination effect was found for the middle 75 mm delamination. In addition, the bottom 75 mm delamination showed a strong delamination effect and had the highest vibration amplitude, as shown in Figure 20c. The vibration amplitude was higher and the vibration pattern was clearer when the delamination was close to the measuring surface (bottom surface). In this case, the size and shape of the delamination could be reliably detected and quantified. Therefore, the experimental results indicate that the depth of the delamination had a more significant effect on the detectability of the delamination than the size of the delamination. 

## 7. Conclusions

In this paper, two composite specimens with different delamination sizes and depths were implemented using NDT and SHM techniques. First, the NDT inspection was used to detect the simulated delaminations in the specimens. The shape, size, and depth of the delaminations could be successfully detected and visualized from the ultrasonic C-scan results. Then, the local defect resonance frequencies of the delaminations were determined from the FFT results of SLDV measurements. The delaminations of different sizes and depths significantly modified the local defect resonance frequency. Finally, the measured operational vibration shapes at the local defect resonance frequencies were extracted from the FEM simulations and experimental measurements. The strong local vibrations only occurred in the delamination regions and the delaminations could be successfully detected and quantified from the measured operational vibration shapes. Moreover, experimental results indicated that the effect of delamination depth on the detectability of the delamination was more significant than the size of the delamination. The experimental and FEM results demonstrated a good capability for the assessment of delaminations with different sizes and depths in composite structures. 

The presented methodology is a baseline-free damage detection technique, as opposed to the conventional Lamb wave SHM methods that involve the comparison of structural response data to a prerecorded baseline taken while the structure is in pristine condition. This methodology can be used for the in-situ detection and quantification of fatigue cracks in metallic plates and debonding in honeycomb sandwich composite structures. In applications, the PWAS transducer needs to be installed close to the damage so that a strong local vibration due to the damage can be excited and detected. For future work, it is desirable that the methodology should be extended to detect and quantify the low-velocity impact-induced delamination in complex composite structures, which is a commonly observed damage in engineering applications. Impact delamination typically occurs with other types of failures, such as matrix cracking and fiber fracture, which makes it difficult to detect.

## Figures and Tables

**Table 1:** Engineering constants of the unidirectional prepreg [5].

*E*_11_	*E*_22_	*E*_33_	*ν*_12_	*ν*_13_	*ν*_23_	*G*_12_	*G*_13_	*G*_23_	*ρ*
140.8 GPa	11.3 GPa	11.3 GPa	0.31	0.31	0.5	5.7 GPa	5.7 GPa	3.4 GPa	1640 kg/m^3^

**Table 2:** Resonance frequencies of different size delaminations in the cross-ply composite plate.

**Delaminations**	25 mm	50 mm	75 mm
**Resonance Frequency (kHz)**	99, 204	28	52

**Table 3:** Resonance frequencies of the delaminations at different depths in the unidirectional [0]_30_ CFRP composite plate.

**Delaminations**	Top	Middle	Bottom
**Resonance Frequency (kHz)**	33, 52	88	35, 62
